# Editorial: ASICs: Structure, Function, and Pharmacology

**DOI:** 10.3389/fphys.2022.831830

**Published:** 2022-01-31

**Authors:** David M. MacLean, Enrique Soto

**Affiliations:** ^1^Department of Pharmacology & Physiology, University of Rochester Medical Center, Rochester, NY, United States; ^2^Instituto de Fisiología, Benemérita Universidad Autónoma de Puebla, Puebla, Mexico

**Keywords:** ASIC, fear conditioning, desensitization, amiloride, pain, immune activation, glioblastoma, cell signaling

The receptor for protons was first reported by Krishtal and Pidoplichko in the early 1980s (Krishtal and Pidoplichko, 1980, 1981). This serendipitous discovery, and the concept of a proton-sensing channel in general, remained controversial for some time (Krishtal, 2015). Eventually, the cloning of ASIC1a clearly established that acid-sensing ion channels exist and ushered in a new era of investigation (Waldmann et al., 1997). In short order, additional ASICs subunits were cloned (or identified) allowing detailed biophysical and physiological work to commence in earnest. These early studies characterized the acid evoked currents from ASICs, established the ionic selectivity and inhibition by amiloride, and ultimately granted ASICs full “citizenship” as proton gated Na^+^ selective channels.

Subsequent to the cloning of ASICs, more selective pharmacological agents such as Psalmotoxin and APETx2 were identified, knockout mice were generated and the first ASIC structure was solved. All of these landmarks spurred the field forward, opening new avenues of inquiry and expanding our knowledge of the physiological and pathophysiological roles these channels play. This expansion of ASIC research is clearly reflected in the literature. A Pubmed search for (https://pubmed.ncbi.nlm.nih.gov/) “Acid Sensing Ionic Channels” either in the title, the abstract or the key words, within the years 1980–2021, shows a clear increase in published papers using this term (Figure 1). The first use was in 1997 in a paper by Waldmann et al. (1997), and the number of papers peaked in 2015 with 70 and has remained around 60 or so per year (Figure 1).

**Figure 1:**
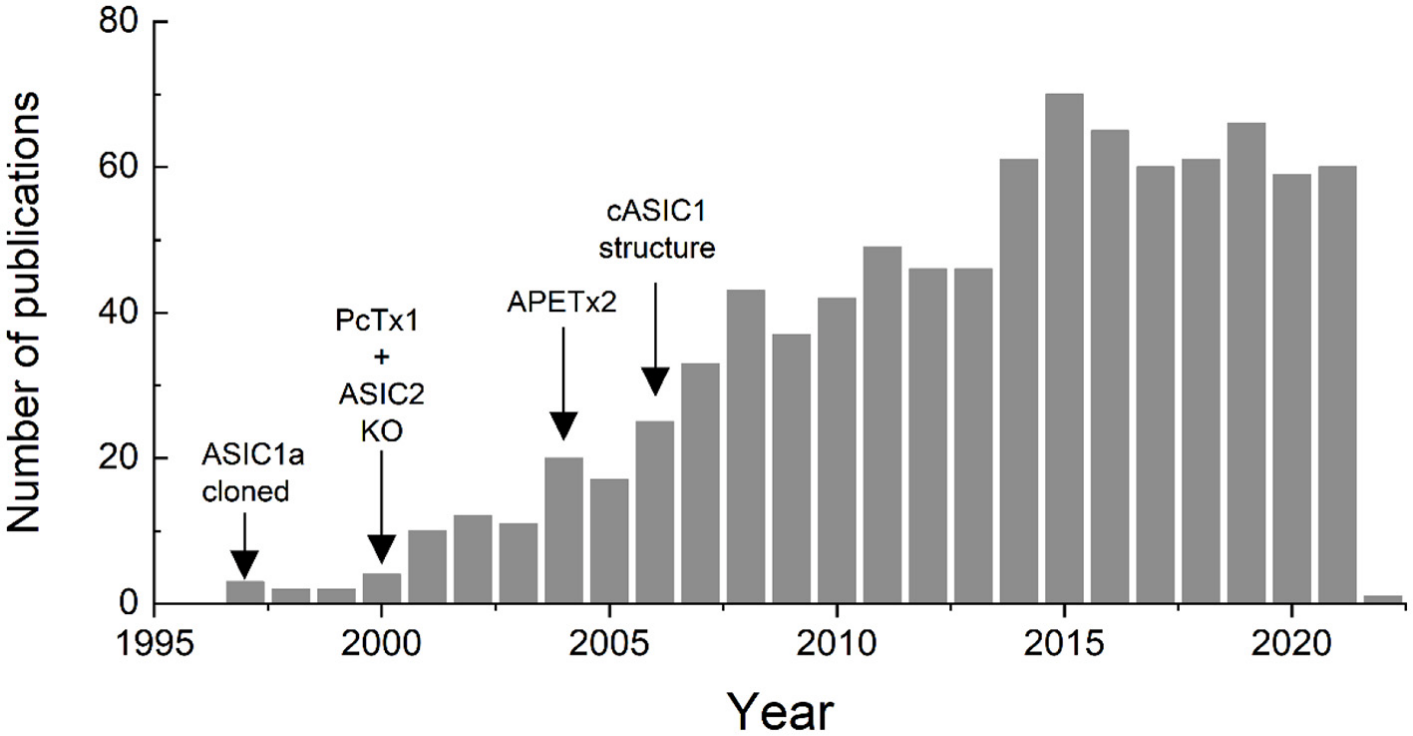
Number of papers published since 1997 in which the term “acid sensing ionic channel” is included in the title, abstract or key words, according to a search performed in Pubmed.

The articles collected in this Research Topic reflect the increasing diversity of the field and the myriad roles played by ASICs. These papers range from basic structural mechanism of desensitization and pharmacological interactions on to ASIC participation in processes such as immune cell activation, sensory coding, fear conditioning and pathological processes such as neoplastic cell migration, epilepsy, and neurodegenerative processes.

This collection features four reviews covering the understudied role of ASICs in the immune system (Foster et al.), in the physiopathology of neurodegenerative processes (Mango and Nisticó), the contribution of ASIC1 to migration of human malignant glioblastoma cells (Shah et al.), and in nociceptive sensory coding (Paéz et al.).

ASICs activate and desensitize quickly. In fact, the original report of proton-gated currents developed a specific method for fast perfusion to resolve these currents (Krishtal and Pidoplichko, 1980; Krishtal, 2015). Despite high resolution structures in multiple functional states, the precise sequence of molecular motions linking the resting or open states with the desensitized state is unclear. Rook et al. focus on this problem of ASIC desensitization and recovery using channel mutagenesis, fast perfusion electrophysiology, and molecular dynamics (Rook et al.). The authors examined the role of the β11/β12 linker that previous observations have shown may be critical for controlling desensitization and recovery of ASIC1 (Rook et al., 2020). They find this linker to be highly sensitive to even small mutations while also uncovering new electrostatic interactions that control desensitized state stability.

Amiloride is a critically useful molecule in the study of the ASICS. While generally used as an ASIC blocker, amiloride can produce potentiation of ASIC responses under certain conditions. The molecular mechanism for this paradoxical effect is unclear. In the work by Matasic et al. the authors used detailed electrophysiological investigation of amiloride effects on ASIC3. They find the potentiating effects largely occur at more alkaline conditions and seem to depend on the presence of external Ca^2+^. Further, they show that disrupting the purported amiloride binding site (G445C) reduces both the inhibitory and potentiating actions, suggesting amiloride exerts these complex actions partly through a shared site.

The role of ASICs in memory consolidation has been a significant subject of research in the field. Fear memories consolidation and retrieval are essential in defining animal behavior. CO_2_ inhalation increases the lability of fear memories during retrieval in mice and this process depends on ASIC1a. In the paper by Taugher et al. the authors report that 10% CO_2_ inhalation soon after fear acquisition increased cued and context fear memory. The effect of CO_2_ was time-dependent (time window of 1–4 h after fear acquisition). CO_2_ inhalation did not alter fear memory in ASIC1a-/- mice thus indicating that ASIC1a activation by acidosis induced by neuronal hyperactivity may enhance memory.

The study by Neuhof et al. found that ASICs, particularly ASIC1a, are expressed in a human mesencephalic cell line (LUHMES) with characteristics of dopaminergic neurons. Using a combination of PCR, Western blot, patch-clamp, and fluorescence imaging techniques, the authors found that ASIC1a is mainly expressed in these neurons and that its blockade reduced neurite outgrowth in developing LUHMES cells. The work is relevant for the understanding of the role of ASIC1a in human pathologies such as Parkinson disease.

An emerging topic of interest is the intracellular signaling cascades activated by ASICs. To gain insight into this question, Salinas et al. investigated how mouse, rat, and human ASIC1a might trigger intracellular signaling via by proton or non-proton stimuli. Using either acidic pH or ASIC-activating toxins at neutral pH (ie. MitTx), they found pERK stimulation to be greater using non-proton activation than by the pH stimulus. Further, this effect was completely absent in ASIC1a knockout mice.

A final paper by Alijevic et al. addresses a particular mystery in the ASIC field. ASICs are activated by rapid pH drops and quickly desensitize essentially completely, with negligible residual current. These channels are clearly implicated in ischemic stroke yet under those conditions the pH changes are likely to be gradual. In prior work, this team used slow pH ramps to better approximate the pathophysiological course of pH change (Alijevic et al., 2020). They found that pH ramps of intermediate speed were best at promoting neuronal spiking. Here they extend this work to mimic the high extracellular K^+^ and low Ca^2+^ expected during ischemia. Under such conditions, the number of action potentials and the firing time increased strongly with acidification accompanied by a change to higher K^+^ and lower Ca^2+^ concentrations. Interestingly the phenomenon was also expressed in ASIC1 knockout mice suggesting other channels may be involved.

It has been more than 40 years since a receptor for protons was first reported. We have come a long way from doubting the existence of proton-gated channels to now having multiple knockout mice, structures, and some pharmacological tools. ASIC research appears in a steady-state or “normal phase” of science in which questions, both big and small, are answered without enormous shifts in understanding (e.g., questioning the existence of the channel itself) (Kuhn, 1970). Some of the remaining big problems include what is the “typical” pH stimulus experienced by a neuronal ASIC? What role do these channels play in the progression of various neuropathological process or pathology in other tissues? And can we leverage our growing knowledge of ASIC biology to inform real world therapeutic options? Addressing these and other problems will weave the role of ASICs into the great tangle of functional relationships between ion channels, transporters, membrane receptors, and messenger molecules that determine cellular excitability, behavior, and pathophysiology. We expect this collection of papers will further contribute to potentiate the ASIC research progress and understanding of the specific role of ASICs in physiology, pathology, and pharmaceutical potential.

## Author Contributions

All authors listed have made a substantial, direct, and intellectual contribution to the work and approved it for publication.

## Funding

ES work was supported by grant from Consejo Nacional de Ciencia y Tecnología (CONACyT), Fronteras de la Ciencia grant No. 1544.

## Conflict of Interest

The authors declare that the research was conducted in the absence of any commercial or financial relationships that could be construed as a potential conflict of interest.

## Publisher's Note

All claims expressed in this article are solely those of the authors and do not necessarily represent those of their affiliated organizations, or those of the publisher, the editors and the reviewers. Any product that may be evaluated in this article, or claim that may be made by its manufacturer, is not guaranteed or endorsed by the publisher.
